# The Advent of MXene-Based Synthetics and Modification Approaches for Advanced Applications in Wastewater Treatment

**DOI:** 10.3390/membranes15120364

**Published:** 2025-11-30

**Authors:** Isha Soni, Monika Ahuja, Pratik Kumar Jagtap, Vinay Chauhan, Savan K. Raj, Prem P. Sharma

**Affiliations:** 1 School of Advanced Chemical Sciences, Shoolini University, Solan 173229, Himachal Pradesh, India; ishsoni@shooliniuniversity.com (I.S.); vinaychauhan@shooliniuniversity.com (V.C.); 2 Department of Chemistry, Government Adarsh College, Barakatullah University, Harda, Bhopal 461331, Madhya Pradesh, India; ahujamonika9988@gmail.com; 3 Department of Chemistry, The ICFAI University, Raipur 490042, Chhattisgarh, India; pratikkumarjagtap@iuraipur.edu.in; 4 Institut National De Recherche Scientifique (INRS), Energie Matériaux Telecommunications, 1650 Lionel-Boulet Blvd., Varennes, QC J3X1P7, Canada; 5 LPPI, Cergy Paris University, 95000 Cergy, France

**Keywords:** two-dimensional, MXenes, water-treatment, forward osmosis, composite membranes

## Abstract

MXenes, members of two-dimensional materials, were discovered in 2011 for the first time. MXenes are famous nowadays for their attractive and unique properties such as hydrophilicity, surface area, and catalytic activity for various industrial applications. This review comprehensively focused on composite membranes with MXenes that can be directly deployed for water purification. Moreover, this review will also give significant insight into new synthetic approaches for MXene-based composite membranes. A review of the utilization of MXene-based composite membranes in modern separation techniques such as nanofiltration, ultrafiltration, and forward osmosis has also been summarized. Finally, the current issues and future perspectives on applying two-dimensional materials for water treatment are elaborately discussed.

## 1. Introduction

The clean water crisis is a global issue that needs to be addressed using sustainable yet energy-efficient membrane-based separation techniques such as UF, NF, and RO [1,2,3]. During the last decade, the interest in the use of membrane technology has gained prominence among researchers owing to the sustainability and recycling of wastewater. Conventional water treatment follows mainly three steps: pre-treatment, actual treatment, and post-treatment [4]. Pre-treatment involves the conditioning of saline water by employing physical, chemical, and biological processes. Large-sized particles like seaweed and debris can be removed using filters directly, and heavy particles can be eliminated by settling down them with gravity [5]. The above-mentioned methods could readily remove macro-sized pollutants, but, still, there are micro-sized contaminates that could require more advanced techniques such as ultrafiltration, nanofiltration, reverse osmosis, etc. [6,7]. Moving ahead with the advanced filtration technologies, nanomaterials have been used as fillers to enhance the performance of the system, as nano-based materials are economically feasible, easy to handle, and bear numerous functionalities for wastewater treatment [8].

One of the most popular membrane-based separation techniques uses MXene as a 2D lamellar material known for its unique properties that are increasingly used to improve membrane performance. MXenes are transition metal carbides and/or nitrides with the general formula of MXene Mn^+1^AXn (n ¼ 1, 2, or 3), where M represents an early transition metal (Ti, V, Cr, Nb, etc.), A represents another element from group IIIA/IVA (Al, Si, Sn, In, etc.), and X represents C and/or N, generated by selectively etching out layers A from the MAX phase and exhibits significant hydrophilicity and selectivity [9,10]. Herein, M layers that are nearly hexagonally close-packed are interleaved with pure A-group element layers and X-atoms. The MXene-based membranes are used in water treatment [11,12] as they exhibit remarkable properties of hydrophilicity, high mechanical strength, and negative surface charge. Good mechanical strength and high-pressure resistance exhibited by MXene-based nanofilters are efficient in the separation of larger particles [13].

Other applications of MXene-based membranes include ion-sieving membranes, solar desalination, and pervaporation desalination [14]. Although the current applications of these MXene-based separation techniques may be limited with more recent properties reported, we expect more research and development in optimizing the mechanical, optical, and electrical properties of this material to explore more and more arenas of sustainable research.

Our goal of this review is to present a summary of the current research and future prospects of MXene-based membranes, such as lamellar membranes and mixed matrix membranes (MMMs), in the viewpoint of separation and purification applications. Moreover, recent advancements focusing on water purification based on the parameters such as water flux and solute rejection have also been covered. In addition, a suggestive outlook regarding future developments on MXene-based membrane research, such as the employment of unexplored membrane configurations to increase working capacity, is provided, which will arouse new ideas for the materials science researchers working on membranes.

### 1.1. Types of MXene-Based Membranes

There are three principal MXene-based membranes that can be fabricated, namely (i) nanostructured membranes in which MXene is used as a base to produce a lamellar membrane, (ii) thin-film nanocomposites (TFNs) in which MXenes are incorporated into the interlayers of a membrane, and (iii) mixed matrix membranes (MMMs) in which the mixing of MXenes with nanomaterials is performed to produce a mixed matrix membrane [15].

#### 1.1.1. Nanostructured Membranes

Nanostructured membranes are lamellar membranes produced by simple procedures such as vacuum-assisted filtering. The first step to create lamellar membranes is to disperse MXene in solvents to form a stable homogenous dispersion [16,17,18]. MXene membrane is fabricated using polyacrylonitrile (PAN) as substrates and Ti_3_C_2_T_x_ are dispersed. In addition, the MXene-based lamellar membranes performance can be improved using silver (Ag), titania (TiO_2_), and ferric hydroxide (Fe(OH)_3_) nanoparticles [19,20,21,22].

#### 1.1.2. Thin-Film Nanocomposites

Thin-film nanocomposites (TFNs) have shown commercial utility as promising membranes for wastewater treatment processes such as forward osmosis, reverse osmosis, etc. TFN membranes consist of an ultrathin polyamide (PA) layer coated over a porous supporting layer of substrate creating an asymmetric structure. Furthermore, the MXene-filled thin-film nanocomposite membranes are created by interfacial polymerization (IP), which is discussed later in the review.

#### 1.1.3. Mixed Matrix Membranes

Polymer-based MMMs are synthesized by integrating filler particles into the polymer solution, so solvent evaporation is carried out to change the phase and cast the membrane [23]. MXene-based materials are polar and hydrophilic, and they can be modified further. In particular, Ti_3_C_2_T_x_ nanosheets have OH ends on the surface that can be functionalized and are chemically stable in polar organic solvents such as NMP, DMF, etc., [24,25] which makes them compatible as fillers useful in making MMMs. MXene fillers have various functions in the synthesis of MMMs, such as their selective solute transport, narrow interlayer spacing, enhanced mechanical properties, high electrical conductivity, and surface area that enhances membrane-based separations. Moreover, MXene-based MMMs have better separation performance owing to their surface chemistries at the filler/polymer interfaces. One of the studies showed that a 1.5 μm thick Ti_3_C_2_T_x_/PVA MMM showed good electrical conductivity and better mechanical stability, thereby showing water fluxes and electrostatically adjusted rejection rates at varying voltages [25,26,27,28].

### 1.2. Fabrication Methods of MXene-Based Polymeric Membranes

**Figure 1:**
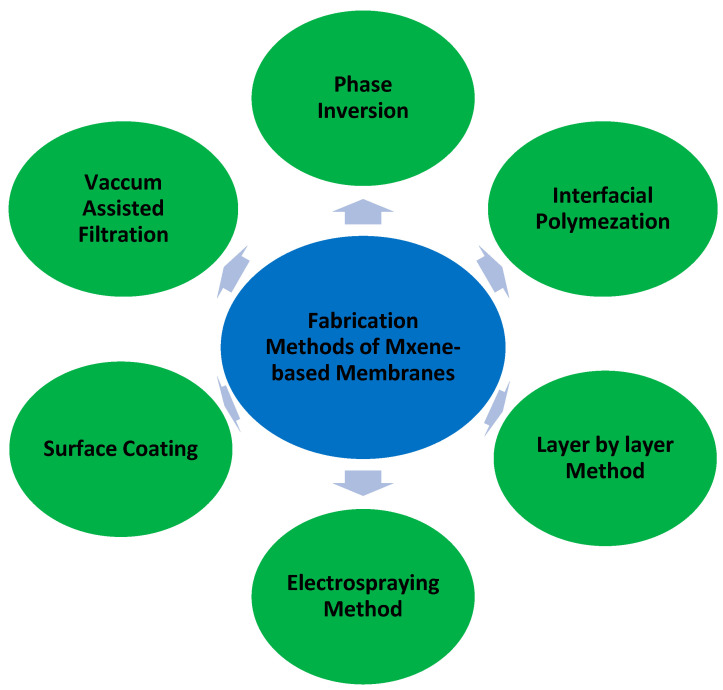
Fabrication methods of MXene-based membranes.

In recent years, several methods have been reported in the literature for constructing MXene-based polymeric membranes [29]. These include phase inversion, vacuum assisted filtration, interfacial polymerization, layer by layer method, electrochemical deposition method, and surface coating, etc., as depicted in Figure 1.

Phase inversion is a well-established technique for researchers to synthesize thin-film membranes. MXene nanosheets are incorporated into the polymeric matrix to form MXene-polymeric membranes. The solvent component is removed from the polymeric solution to synthesize the membrane. MXene is used to augment rejection rate, improve pore morphology, and amplify total flux [30].

Vacuum-assisted filtration is one of the most cost-effective methods to produce MXene membranes. An appropriate amount of MXenes and polymer are amalgamated together in a polar solvent, and the final dispersion is obtained after sonication. After that, filtration using a vacuum pump is carried out to form a thin layer on top of an ultrafiltration substrate. The obtained MXene nanosheets can form nanochannels that allow water to permeate while rejecting other molecules [31,32].

Interfacial polymerization (IP) is another well-known technique to prepare nanocomposite membranes. This synthesis process includes the reaction of two monomers with each other in an immiscible solvent. The first step in this technique includes coating an ultrafiltration substrate, which is dried and soaked in an organic solution [33]. After that, nanomaterial MXene is added in the initial phase and loaded on the polymeric membrane to restrict salt and dye molecules.

Another useful method is the layer-by-layer method which forms uniform multi-layered membranes with oppositely charged molecules. In this technique, the substrate is made to develop either a positive or negative surface charge. For instance, Ti_3_C_2_T_x_ and poly(diallyl dimethylammonium chloride) (PDAC) membranes were prepared in one study using a layer-by-layer approach. The layer formation depends on hydrogen bonding or electrostatic attraction between oppositely charged layers [34,35].

One more commonly used technique is the electrospinning method in which highly porous nanofibrous membranes are produced. In this technique, electrospun membranes are synthesized with uniform pore size that have porosities > 90%. These membranes are useful in the pre-treatment of water to remove divalent ions and oil molecules [36]. Electrically conductive MXene/polymeric membranes are synthesized by electrochemical deposition in which MXenes are deposited on the polymeric substrate via electrolysis [37].

## 2. Applications of MXene-Based Membranes in Water Treatment Methods

### 2.1. Ultrafiltration

**Figure 2:**
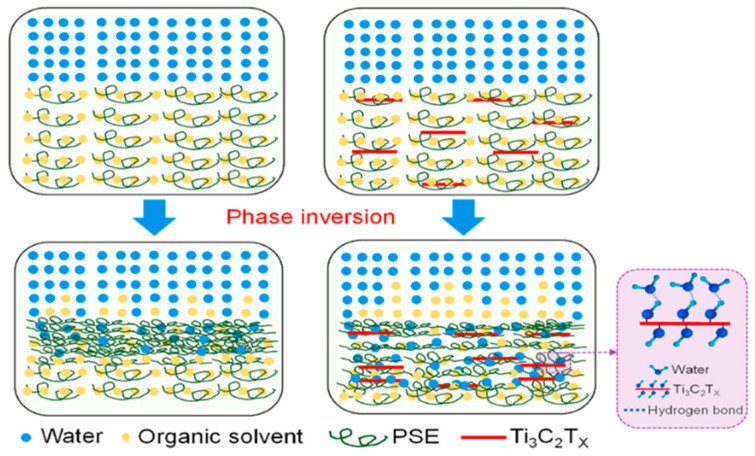
Phase inversion procedure for the PSE casting solution with and without dopant—Ti_3_C_2_T_X_ nanosheets [41]. Reproduced with permission. Copyright 2021, Publisher Elsevier.

The UF membrane (UFM) functions at specific values of pressure, providing economical and effectual separation, and involves removing wasted powdered components from feedwater, in contrast to NF and RO membranes. Although ultrafiltration (UF) has been broadly applied in water treatment, membrane fouling remains a major problem to be addressed [38,39]. In order to improve its anti-fouling qualities or create an entire UFM, UF-MXene nanocomposite membranes are employed in the phase inversion in situ filling techniques. Moreover, a smaller water contact angle of MXene has been further reported to enhance the hydrophilicity of the traditional nanocomposite membranes. According to Rasool et al. [26], all altered membranes had remediation rates above 90% compared to the unaltered polysulfone membrane’s 77.48% rejection rate for bovine serum albumin. Better flux regaining ratios and the reversible fouling reported are believed to enhance the MXene concentration. Membranes with different thicknesses (0.6 to 1.8 mm) are created by applying a layer of 2–6 mg of Ti_3_C_2_T_x_ nanolayers to a 47 mm caliber polyvinylidene difluoride platform. The contact angle with water reported for polyvinylidene difluoride is 81° which cause rejection due to its hydrophobicity. Further coating of the membrane with Ti_3_C_2_T_x_ nanoparticles decreases the angle to 37°, indicating that the surface of the membrane became extremely water-repellent. The surface properties of the membrane were further reduced as the MXene content increased. This phenomenon results from a higher density caused by a larger assembly of MXenes in the prominent top sheets of the membrane. A change in the cross-sectional design of the membrane can speed up the interchange of solvents and water molecules throughout the phase inversion procedure, which is influenced by MXene’s hydrophilic characteristics [40]. The potential mechanism of integrating Ti_3_C_2_T_x_ into the membrane’s polymeric structure was demonstrated by Zhou et al. [41]. The Ti_3_C_2_T_x_ nanosheets’ hydrophilic properties are essential for improving water transport. This is accomplished by hydrogen bonding, which speeds up phase inversion, thins the topmost layer, and further encourages the creation of voids inside the membrane arrangement. Additionally, the Ti_3_C_2_T_x_ nanosheets’ hydrophilic and thin properties help to significantly lower the doping composition to abnormally low levels. Figure 2 shows the phase inversion procedure for the polysulfate (PSE) casting solution with and without Ti_3_C_2_T_x_. Very tiny amounts of Ti_3_C_2_T_x_ nanosheets added are reported to enhance phase separation and increase the pore volume of the membranes created.

**Figure 3:**
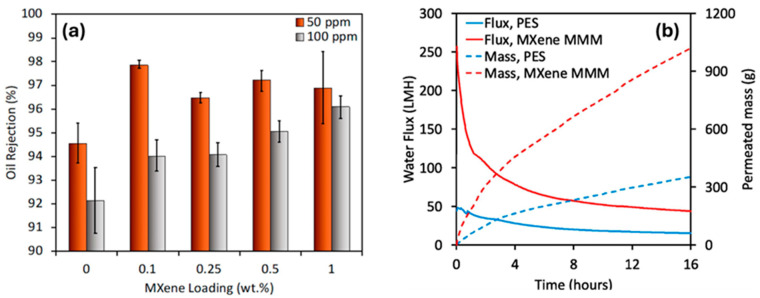
(**a**) Oil remediation of 50 and 100 ppm oil–water emulsion performed at 1 bar pressure, (**b**) shows the emulsion flux and permeated mass carried up to 16 h [47]. Reproduced under CCBY license. Copyright 2024, Publisher Elsevier.

In order to improve solvent dehydration, separate 2D Ti_2_CT_x_MXene membranes were created using a hyperbranched polyethyleneimine-based aluminum oxide platform (anode) with a pore size of approximately 100 nm [42]. The overall performance of the membrane may be adversely affected by this observation [43]. The membrane quality was further improved by employing a direct interfacial polymerization technique. Using this method, the hyperbranched polyethyleneimine that was first added to the membrane reacted with the trimethyl chloride. Effectively sealing all possible non-selective flaws was the goal [44]. In another project, MXene-UF was used to remove dye from aqueous media [45]. Using wastewater containing synthetic dyes as the feed solution, MXene-UF composite along with the ground activated carbon (PAC)-UF were tested to evaluate their performances. To mimic situations that are frequently encountered, dyes, specifically methyl orange (MO) and MB, were selected. While the pristine UFM displayed normal flow (0.86 for MB and 0.90 for MO), MXene-UF performed better than single UF (with 0.90 for MB and 0.92 for MO) and PAC-UF (with 0.72 for MB and 0.75 for MO). As opposed to UF, the hybrid system’s incorporation of an adsorbent reduced the inevitable fouling caused by the dye removal. ZnFe_2_O_4_-MXene nanocomposites were produced by precipitation in order to improve the permeability and anti-fouling characteristics of polyether sulfone (PES) UF membranes. These nanocomposites were further combined with PES to create mixed matrix membranes (MMMs) that had excellent permeability and anti-fouling properties [46]. The report showed that ZnFe_2_O_4_ accumulation enhanced the overall ZnFe_2_O_4_-MXene nanocomposites’ dispersion within the PES, promoting significant material interconnection on the Ti_3_C_2_T_x_ laminates’ surface as well as within the interlayer structure. The hydrophilic groups present on the ZnFe_2_O_4_-MXene nanocomposites’ surface further increased hydrophilicity, pore design, and surface polarity of the MMMs. In another reported study, Ahmed Nabeeh et al. [47] intercalated Ti_3_C_2_T_x_ nanoparticles into a PES matrix to create MMMs and demonstrated an ultra-high water flux of 2280 LMH/bar, 98% oil remediation and a water vapor permeance (WVP) of 18,100 GPU (Figure 3a,b). In comparison to the control, the MMM exhibited a 50% decrease in irreversible fouling, as reported in the anti-fouling testing conducted utilizing a 1000 mg/L milk solution.

**Figure 4:**
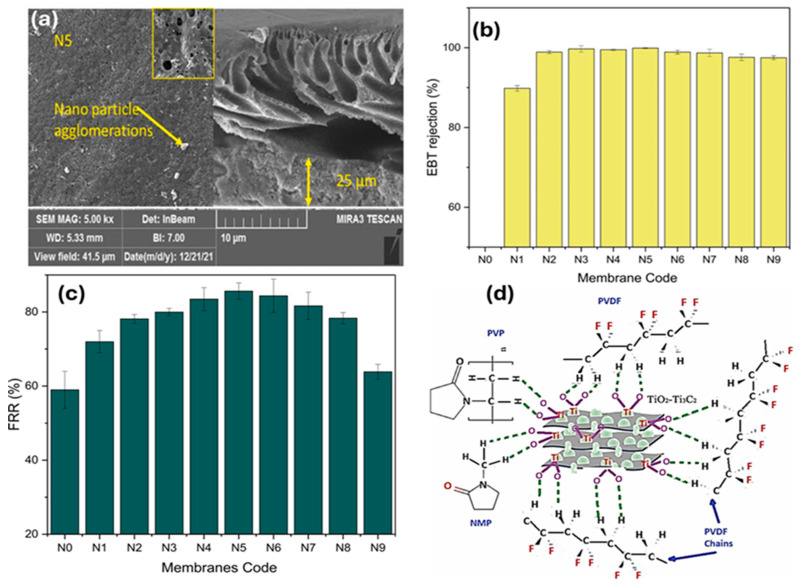
Images captured from (**a**) FE-SEM of the synthesized PVDF/MXene membrane, (**b**) EBT remediation of the pristine PVDF and modified membranes with MXenes, (**c**) water flux recovery ratio membranes, and (**d**) the plausible interaction mechanism of the prepared membrane [48]. Reproduced under CCBY license. Copyright Elsevier 2024.

For textile wastewater treatment applications, Abood et al. [48] conducted a study that presented a unique asymmetric PVDF-based ultrafiltration membrane that had been bulk modified with MXene to further increase its fouling resistance (Figure 4a). For the Eriochrome Black T (EBT) dye solution, the produced membranes showed a flow of 467.8 LMH and a maximal water flux of 538 LMH. Additionally, the composition of the nanocomposite membrane was optimized, resulting in total remediation of EBT (Figure 4b). A steady increase in the flux recovery ratio (FRR%) for the MMMs, made from 0.5 weight percent MXene, reached a high value of 85.6% (Figure 4c). Additionally, physical coordination among the membrane elements was discussed further in the mechanism (Figure 4d).

### 2.2. Forward Osmosis

**Figure 5:**
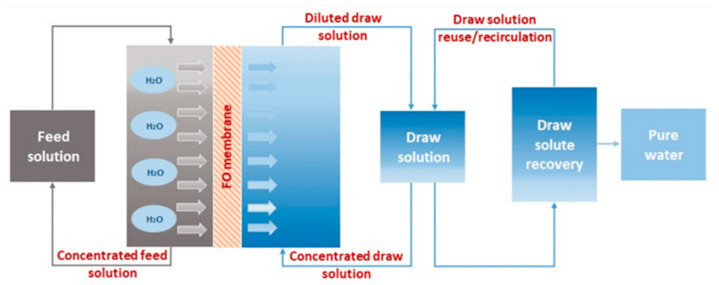
Schematic diagram showing forward osmosis for water purification [49]. Reproduced under CCBY-license. Copyright 2023, Publisher MDPI.

Since an osmotic pressure gradient, rather than hydraulic pressure, transports water across the membrane from the feed solution to the draw solution as shown in Figure 5, forward osmosis can be operated at a low expense. The forward osmosis process provides high water flux, enhanced adaptability, minimal system energy utilization, and reduced fouling of the membranes. Nevertheless, the forward osmosis process also has numerous barriers. Out of them, the two primary issues are relevant membrane selection and drawing solution [49]. Two-dimensional MXene membranes are an excellent option for forward osmosis owing to their high surface area, high water flux, and improved permeability, as reported earlier [50,51,52].

Ascorbic acid is incorporated into the layers of Ti_3_C_2_T_x_MXene and aluminum ions are used as a cross-linking agent in the MXene membrane used for drinking water treatment [53]. 

The stability of the ascorbic acid-modified MXene membrane was evaluated using antioxidant activity and zeta potential values, which confirmed that the presence of ascorbic acid increased the lifetime of the MXene membrane up to 30 days. The life span of the MXene membrane and ascorbic acid, and the MXene membrane and ascorbic acid and Al^3+^ was even studied using surface morphology analysis, which indicated that the MXene membrane and ascorbic acid, and the MXene membrane and ascorbic acid and Al^3+^ do not show dry cracks and defects, as shown by the MXene membrane after 15 days. This indicated that the ascorbic acid provided stability and enhanced the life of the MXene. The interaction between the MXene membrane and ascorbic acid has been studied using DFT, which shows that ascorbic acid completely adsorbs on the surface of the MXene membrane. During forward osmosis, permeation rates of alkali metal chlorides (NaCl and MgCl_2_) were studied for MXene membrane, MXene membrane and ascorbic acid, MXene membrane and ascorbic acid and Al^3+^, and even Al^3+^ and graphene oxide. Out of them all, the MXene membranes and ascorbic acid and Al^3+^ showed the lowest penetration rate for salts. The incorporation of ascorbic acid and Al^3+^ into the MXene membrane enhanced the osmotic inhibition rate of NaCl to 53.84% and MgCl_2_ to 58.26%. In a similar study, a composite membrane was prepared by Mu et al. [54], wherein different ratios of dopamine and polyethylene glycol (10:0, 3:1, 1:1, 1:3, 0:10) were introduced into the layers of the MXene membrane by the filtration method. According to surface analysis, the uniform distribution of particles was seen in the case of the 1:1 MXene membrane, while in others, agglomeration was seen to have happened. This 1:1 MXene membrane possesses a similar d-spacing value as that of the MXene membrane (13.50 Å) but a reduced contact angle of 62.990°, thereby increasing the hydrophilicity of the composite formed. This showed resistance to the flow of water molecules and a reduction in durability. Different pollutants such as Coomassie Brilliant Blue R250 and methylene blue at 0.5 MPa 1:1 MXene membrane gave rejection rates of 94.2% and 82.4%, respectively, using filtration tests. For all of the ions, the membrane showed a rejection rate of >98% in the hour test in an H-shaped cup (having 0.2 M, 50 mL solution on both sides separated by a membrane). However, a greater permeation was found towards Na^+^ than Mg^2+^ due to the greater size. The membrane was tested for permeance for 38 days and maintained a permeance of 750 Lm^−2^h^−1^. The addition of biomolecules enabled the increase in d-spacing values of the membranes, giving more durability for permeance.

**Figure 6:**
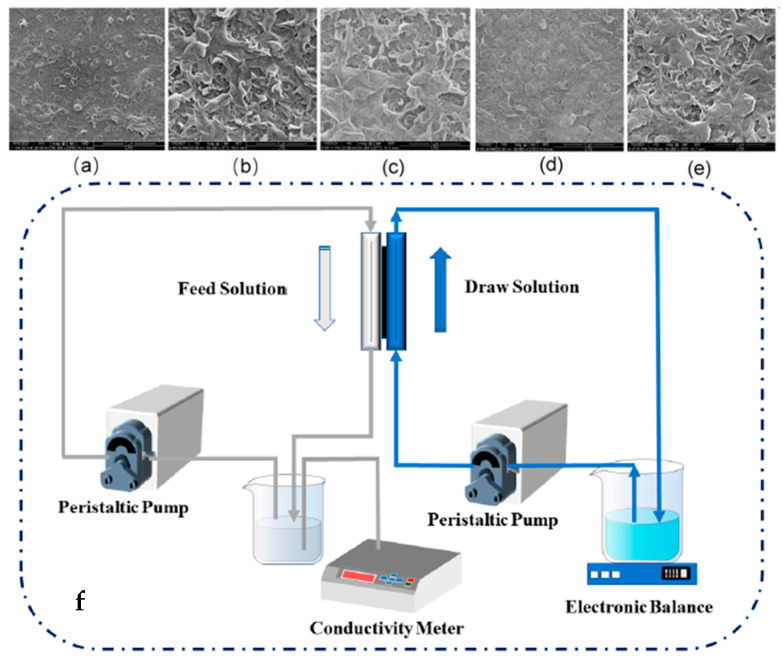
SEM images of (**a**) T-1, (**b**) T-2, (**c**) T-3, (**d**) T-4, and (**e**) T-5 at 5 μm scale. (**f**) Experimental setup of reverse osmosis. [56]. Reproduced under CCBY-license. Copyright 2022, Publisher MDPI.

**Figure 7:**
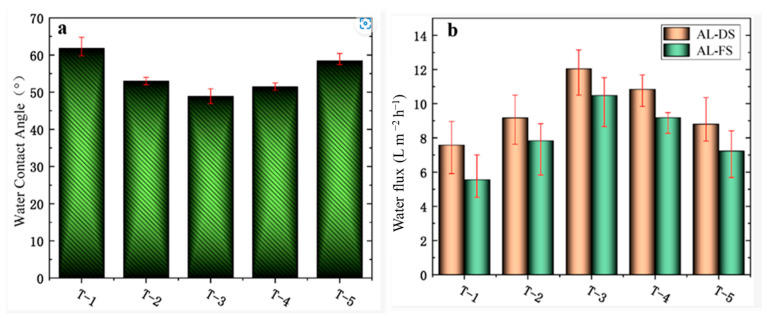
(**a**) Water contact angle and (**b**) water flux comparison for T-1, T-2, T-3, T-4, and T-5. (AL-DS: active layer facing draw solution and AL-FS: active layer facing feed solution) [56]. Reproduced under CCBY-license. Copyright 2022, Publisher MDPI.

Further preventing organic fouling of the membrane, vacuum filtering of different amounts of MXene (0.18 to 1.09 g/m^2^) has been performed on the polyether sulfone membrane and interfacial polymerization [55]. A membrane with 0.47 g/m^2^ of MXenes showed a minimum resistance of 46.8 Ω when compared to a polyether sulfone membrane (2.1 × 10^12^ Ω) and better water flux with reverse solute flux. The highest decline in water flux was shown by the bare polyether sulfone membrane (12.6%) while the presence and absence of the external electric field of the MXenes and polyether sulfone membrane showed a decline of only 4.5% and 8.2% water flux, respectively. Here, the MXenes prevented the fouling of the membrane caused by organic molecules such as sodium alginate and bovine serum albumin both in the presence and absence of an electric field, to varying degrees. Polysulfone and polyvinylpyrrolidone were employed to prepare forward osmosis membranes with different amounts of MXene (0, 0.005, 0.007, 0.01, and 0.03%) named T-1, T-2, T-3, T-4, and T-5 [56]. SEM images as shown in Figure 6a–e indicate that an increase in the amount of MXene morphology significantly becomes gentle and dense. But the contact angle did not show any specific trend as indicated by Figure 7a and showed the order: T-1 (61.7°) > T-5 (58.02°) > T-2 (53.02°) > T-4 (51.49°) > T-3 (48.92°). However, the highest contact angle was found for the unmodified membrane. This may be ascribed to the agglomeration of MXene particles. The setup employed for water flux using these modified membranes is shown in Figure 6f, and water flux efficiency is shown in Figure 7b. The efficiency results have shown that increasing the amount of MXene up to 0.007% showed that an increase in hydrophilicity is mainly responsible for higher reflux, and (0.01 and 0.03%) agglomeration of MXene results in a decrease in flux. Apart from this, comparing the water flux of the T-3 membrane with other doped polyether sulphone membranes this membrane gave satisfactory results as shown in Table 1.

**Figure 8:**
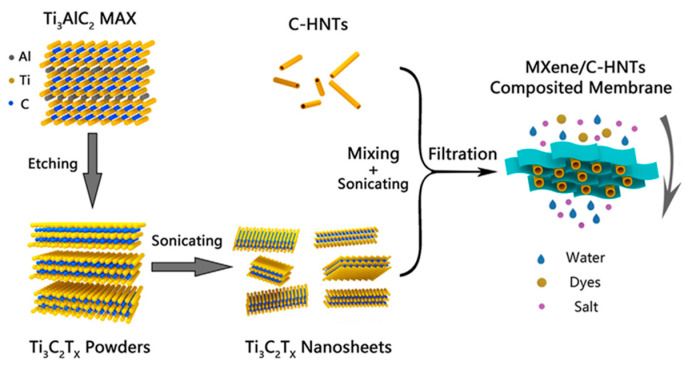
Preparation of halloysite nanotube calleted MXene-based composite forward osmosis membrane [62]. Reproduced with permission. Copyright 2022, Publisher Elsevier.

**Figure 9:**
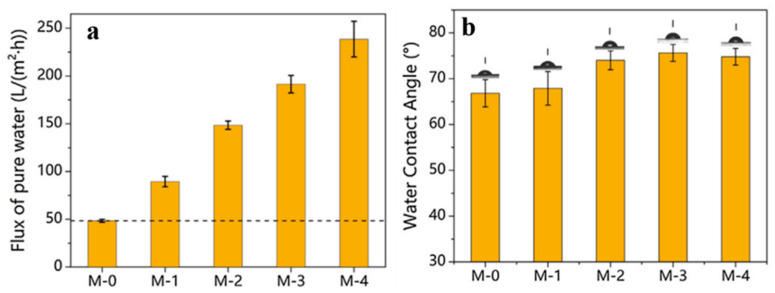
(**a**) Flux of pure water and (**b**) water contact angle comparison for different ratios of MXenes named M-0, M-1, M-2, M-3, and M-4 [62]. Reproduced with permission. Copyright 2022, Publisher Elsevier.

Nanotubes-modified MXene composite forward osmosis membranes have been synthesized as shown in Figure 8 [62]. Herein, 0.72 mL of MXene has been mixed with varying amounts of surfactant (cetyltrimethylammonium bromide), modified halloysite nanotubes (C-HNT) (0, 0.72, 1.44, 2.16, and 2.88 mL) named as M-0, M-1, M-2, M-3, and M-4. The nanotubes helped in increasing the interlayer spacing and provided mechanical strength, and an increase in roughness was observed with an increase in nanotube concentration. The M-4 membrane showed uneven distribution and agglomeration; therefore, M-3 was chosen for further studies. Figure 9 shows that the contact angle increased up to the M-3 membrane and then remained almost constant, but the pure water flux tends to increase from M-1 to M-4. It can be observed in Figure 9a (dashed line), that the fluxes of composite membranes increased continuously with increasing C-HNTs loadings, which was nearly a 4-fold increase. From M-1 to M-3, the increase was found to be 48.4 to 191.4 Lm^−2^h^−1^, while rejection rates for different dyes (methyl orange, rhodamine B, crystal violet, Evans blue, and Congo red) remained >99%, while the rejection rate decrease for different salts followed the trend: NaCl (<1%) < MgSO_4_ (3.3%) < Na_2_SO_4_ (5.7%). Overall, MXene shows satisfactory osmotic separation performance with lower solute reflux.

### 2.3. Membrane Distillation

Operationally, membrane distillation is based upon mass transfer, or thermally induced vapor transport, across a non-wetted water-resistant membrane. Thus, the hydrophobicity of the microporous membrane plays a major role in liquid infiltration while allowing vapor to pass through. The difference in temperature created by the hot feed and cold permeation creates a pressure gradient that runs the vapor diffusion [63]. The membrane serves a dual function, maintaining the pressure gradient as well as a passage for the vapor. Although membrane distillation provides a more cost-effective and energy-efficient operation compared to traditional distillation methods, its large-scale commercial application is hindered due to low productivity (excessive thermal energy consumption, increased temperature polarization, and diminished permeate flux). MD is superior to other membrane-based processes in high-salinity feeds, but inorganic fouling, or scaling, becomes a serious problem that can obstruct the process if ignored. Furthermore, in terms of scaling, wetting, and fouling, these membranes behave similarly to other reverse osmosis membrane systems [64].

To improve its efficiency and minimize thermal energy input, MD has been coupled with photothermal and electrothermal conversion materials. Chemically etched single-layered and multilayered Ti_3_C_2_T_x_MXene membranes were analyzed using morphological techniques and found 0.3 µm interlayer spacing with a lateral dimension of 3 µm [65]. Ongoing from single layer to multilayer, the d-spacing value decreased from 12.636 to 10.109 Å. As per optical analysis, the single-layered MXene fluids displayed better adsorption capacity than multilayered MXene fluids due to the large specific surface area. Employing equal volumes of MXene (0.04 vol%) for the evaporation efficiency and evaporation rate, the single-layered MXene (327.9%, 1.335 kgm^−2^h^−1^) outperformed multilayered MXene (298.4%, 1.243 kgm^−2^h^−1^). Similarly, in the solar distillation experiment, single-layered MXene outperformed with a flux value of 2.375 gm^−2^h^−1^ (feed temperature of 32° C, ΔT = 17 °C), which is far greater than the MXene-based solar evaporator.

**Figure 10:**
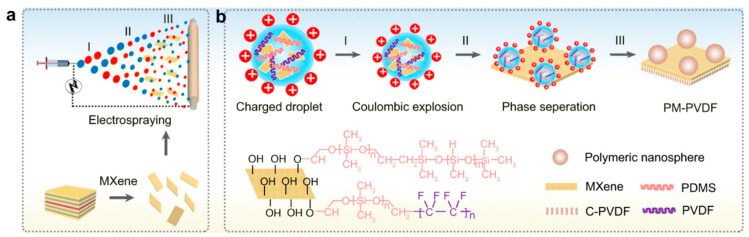
(**a**) Interfacial fabrication for preparation of PM-PVDF membrane; (**b**) incorporation of nanospheres of polydimethylsiloxane at the surface Ti_3_C_2_T_x_MXene and C-PVDF using electrospray method [66]. Reproduced under CC-BY license. Copyright 2022, Publisher Springer Nature.

The incorporation of nanospheres of polydimethylsiloxane at the surface of Ti_3_C_2_T_x_MXene and C-PVDF is achieved by the electrospraying method, as shown in Figure 10 [66]. The electrospray engineering process mainly involves three steps: (I) formation of charged droplets, (II) coulombic explosion, and (III) phase separation. This polymeric nanoassembly was confirmed by SEM and TEM. The MXene presence reduced the average diameter of polymeric spheres from the micrometer to nanometer range with an increase in contact angle from 70° to 172° and a slide angle of <2°, indicating a highly hydrophobic surface for easy passage of vapors. Further, this modification altered the surface porosity from 69.2 ± 0.2% to 71.8 ± 0.3%, which is beneficial for thermal resistance and enhanced thermal membrane conduction. The wetting resistance of the membrane was studied using electrochemical impedance, which shows stable resistance when studied over a gap of 2 h for a period of 24 h. The feed was operated at 30 °C for membrane distillation and gave a freshwater flux of about 1.55 kgm^−2^h^−1^ without solar exposure. With 1 sun illumination, the freshwater production enhanced to 2.88 kgm^−2^h^−1^, but no improvement has been observed in the case of C-PVDF. This showed that the presence of 2D MXene acted as a localized solar harvester and enhanced electrical conductance.

Similarly, PVDF and MXene nanofiber membranes have been synthesized using electrospinning and blending [67]. As the amount of MXene increases from 0 to 74.14 mg, the contact angle decreases, implying an increase in hydrophobicity of the membrane formed, but the contact angle drops slightly as the concentration reaches 112.71 mg. For the evaporation under the Xe lamp and 1 sun illumination, 74.14 mg MXene containing membrane gave the highest evaporation rate of 1.56 kgm^−2^h^−1^ within 60 min. Thus, these results pave the way for its high efficiency in membrane distillation for wastewater treatment.

**Figure 11:**
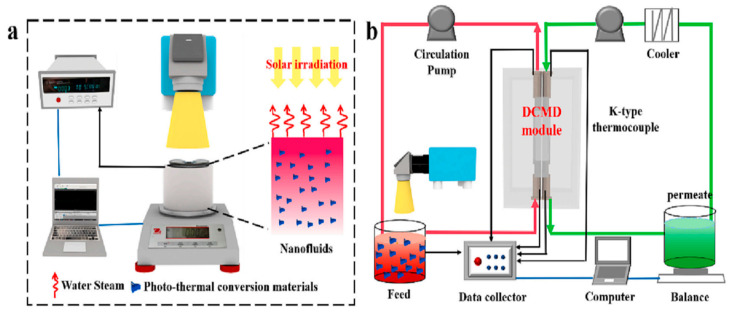
Experimental setup: (**a**) evaporation, (**b**) solar membrane distillation [68]. Reproduced with permission. Copyright 2023, Publisher Elsevier.

Another photothermal membrane distillation process has been studied by employing an iron oxide-modified MXene composite prepared by the reduction approach in a solvothermal process [68]. Surface morphological analysis demonstrated that the uniformly smaller spherical particles of Fe_3_O_4_ accumulated at and between the layers of the MXene. Further, XRD revealed that the interlayer spacing of the Fe_3_O_4_ + MXene composite widened in comparison to the MXene. The evaporation and membrane distillation setups are shown in Figure 11. The 0.02 vol% Fe_3_O_4_ + MXene composite gave an evaporation rate of 0.841 kgm^−2^h^−1^, which is greater than Fe_3_O_4_ (0.570 kgm^−2^h^−1^) in 3.5 weight% NaCl, while the membrane distillation experiment with a feed liquid (0.02 vol% Fe_3_O_4_ + MXene composite) at 35.5 °C with one solar illumination gave a flux of 2.26 kgm^−2^h^−1^ and about 1.28 kgm^−2^h^−1^ flux without solar illumination but a constant membrane temperature difference of 15 °C.

**Figure 12:**
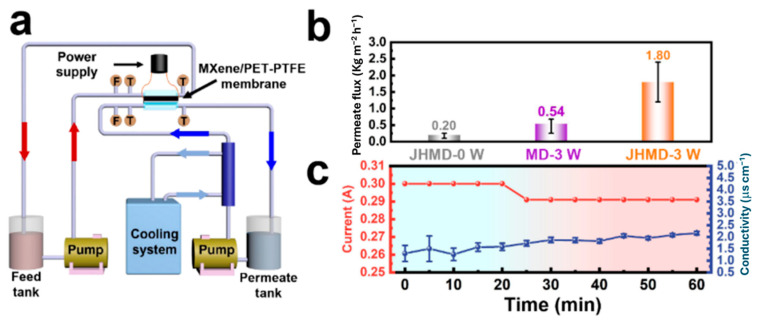
(**a**) Schematic diagram representing membrane distillation; (**b**) comparison of flux at different power inputs for joule heating membrane distillation and conventional distillation using PET-PTFE + MXene nanosheets membrane; (**c**) current flow and conductivity during the joule heating membrane distillation [69]. Reproduced with permission. Copyright 2023, Publisher Elsevier.

Apart from photothermal conversion materials, electrothermal materials have also been explored for membrane distillation. PET-PTFE with MXene nanosheets has been prepared using a vacuum filtration technique and employed as an electrothermal material [69]. This synthesized material showed a decrease in contact angle from 101.92° to 79.90° and a decrease in porosity from 53.84 to 50.31% when compared to PET-PTFE alone. This prepared membrane reached a steady state in 20 min, delivering a rate of 3.17 kgm^−2^h^−1^ at 3 W and 4.53 kgm^−2^h^−1^ at 4 W. This shows that the membrane possesses high vapor generation capacity. The distillation behavior of the membrane has been evaluated using the setup shown in Figure 12a. At 3 W input, 1.80 kgm^−2^h^−1^ flux is generated, which is far higher in comparison to conventional membrane distillation setups wherein a flux of about 0.54 kgm^−2^h^−1^ is achieved due to enhanced current transfer, as indicated by Figure 12b,c. Here, MXene provided extra hydrophilicity, while the low thermal conduction of PTFE allowed high energy efficiency.

### 2.4. Reverse Osmosis

Reverse osmosis (RO) is the latest membrane technology, which is widely used globally for its high efficiency. It uses a semipermeable membrane where water flows in the reverse concentration gradient direction (i.e., from the concentrated to the dilute side). This effective membrane has the capability to typically be used in these membranes. Reverse osmosis (RO) is a highly effective and versatile semipermeable membrane-based water purification technology used to remove a broad range of contaminants such as large molecules and tiny particles, as well as monovalent ions from wastewater, thus providing clean water for various purposes [70]. RO membranes possess very small pore sizes in the range of 0.0001–0.001 μm that block most small organic/inorganic molecules like polymeric materials, such as cellulose acetate, cellulose diacetate, or polyamides, to remove dissolved salts, metal ions, minerals, contaminants, microorganisms, etc., allowing only molecules of water to pass through it [71,72]. RO is a highly promising method used in the desalination of seawater, groundwater, and drinking water production [73]. RO operates via a separation mechanism in which external hydraulic pressure is applied against natural osmotic pressure to overcome a concentration gradient that forces the water to pass through the membrane, leaving behind impurities [70].

The advantages of the RO membrane have garnered noteworthy attention as a promising material for water desalination due to its user-friendly nature, outstanding separation efficiency, and high water permeability [74]. Polyamide (PA) material has been used in RO membranes until now. However, certain drawbacks to these RO membranes exist, such as insufficient hydrophilicity, permeance, and selectivity [75]. Moreover, the membranes tend to have rough surfaces and are vulnerable to oxidation and contamination by a foulant [76]. Recently, to improve the performance of RO membranes’ permeability, chemical resistance, and selectivity [77], various in situ surface modification methods have been adopted such as the incorporation of nanomaterials into PA membranes [78] including surface coating [79] and hydrophilic nanoparticles, e.g., oxides [80,81], graphene [82,83], and carbon nanotubes [84].

MXene is incorporated into the polyamide (PA) layers to enhance the performance and desalination efficiency of reverse osmosis membranes, but an excess amount of MXene added during the modification process could potentially lead to a looser membrane structure and weaker bonding within the material, ultimately influencing the salt rejection capabilities leading to limited potential of MXene in improving the performance of reverse osmosis membranes [85].

**Figure 13:**
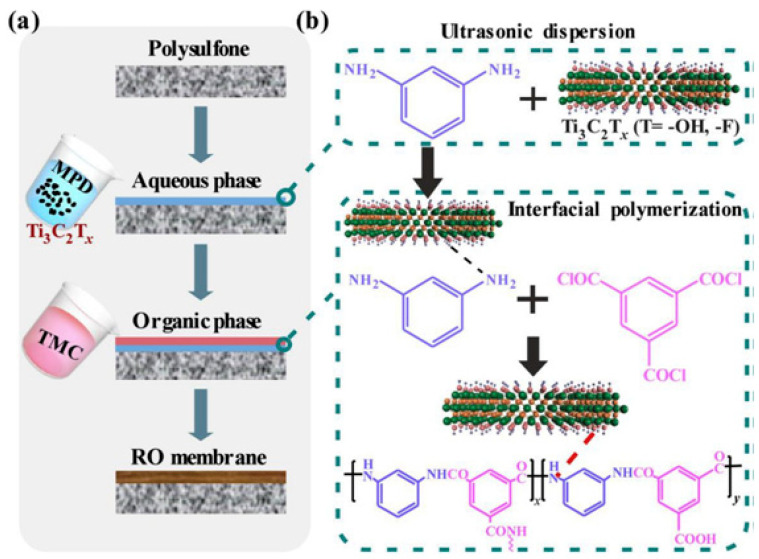
(**a**) Illustration of Ti_3_C_2_T_x_ incorporation into a polyamide reverse osmosis membrane; (**b**) interfacial polymerization reaction mechanism [85]. Reproduced with permission. Copyright 2020, Publisher Elsevier.

**Figure 14:**
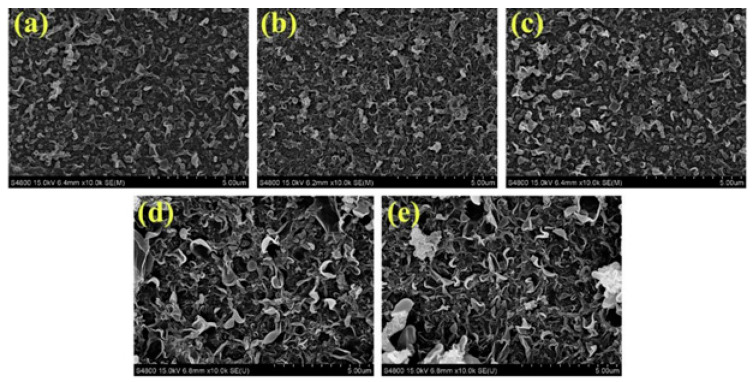
Surface SEM images of (**a**) PA, (**b**) PA05, (**c**) PA10, (**d**) PA15, and (**e**) PA20 membranes (at 5 μm scale) [85]. Reproduced with permission. Copyright 2020, Publisher Elsevier.

In recent years, modifications of RO membranes using 2D nanomaterials, e.g., Ti_3_C_2_T_x_MXene, are of great importance owing to the chemical stability and high surface area of MXene materials. An in situ interface polymerization technique is used to integrate 2D Ti_3_C_2_T_x_ nanosheets (0.01% to 0.02%, fraction of mass) into polyamide thin-film (PA)-RO membranes [86]. The incorporation of layered Ti_3_C_2_T_x_ has shown outstanding characteristics such as augmenting the membrane surface’s hydrophilicity, reducing its roughness, and enhancing membrane endurance and efficiency of desalination under diverse operating conditions [87,88,89,90]. Wang et al. have reported a novel PA-Ti_3_C_2_T_x_ nanocomposite RO membrane made by in situ polymerization with trimesoyl chloride and m-phenylenediamine aqueous solution. Figure 13a shows the representation of 2D MXene Ti_3_C_2_T_x_ implanted into the polyamide (PA) membrane. In addition, Figure 13b shows the composite membrane formed with the PA, Ti_3_C_2_T_x_, and m-phenylenediamine with separating layers bonded by hydrogen bonding and van der Waals forces. Moreover, the experiments were carried out on optimizing pH and reaction time as demonstrated in Figure 14 to guarantee an excellent separation performance [85]. Results showed that the PA-Ti_3_C_2_T_x_ membrane exhibited higher water permeability, ranging from 2.3 to 2.5 L m^−2^h^−1^bar^−1^, as compared to the bare PA membrane, which had 1.7 L m^−2^h^−1^bar^−1^. On the other hand, the high salt rejection (97.9–98.5%) was observed demonstrating the great features of Ti_3_C_2_T_x_ in inhibiting the trade-off effect [91,92]. This resulted due to the -OH groups produced by the Ti_3_C_2_T_x_ and amplified cross-linking of the PA-Ti_3_C_2_T_x_ membrane selective layer.

Simultaneously, the PA-Ti_3_C_2_T_x_ membrane has shown an enhanced anti-fouling, and chlorine resistance outperforming the PA membrane and other RO membranes in the literature. Studies reported after a chlorine-resistance test at 10,000 ppm h, a high salt rejection of 97.1% for the PA-Ti_3_C_2_T_x_ membrane was still retained [91]. Based on the observed microstructures and mechanism analysis, chlorine resistance was mainly attributed to the interaction between surface functional groups of Ti_3_C_2_T_x_ nanosheets with active chlorine. Thus, various literature studies demonstrated the use of Ti_3_C_2_T_x_ incorporated water desalination membranes to improve the performance of water treatment systems was advantageous for practical applications.

### 2.5. Nanofiltration

**Figure 15:**
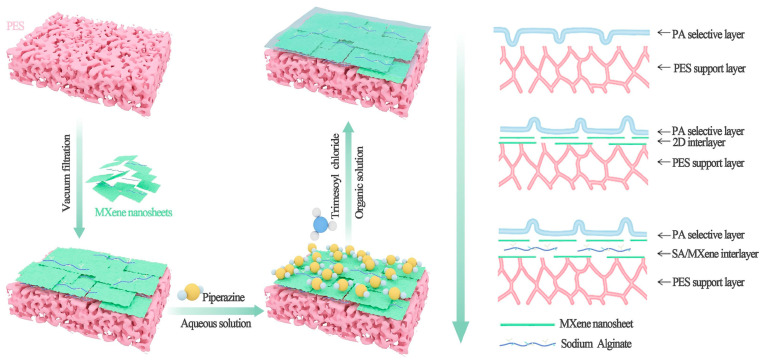
An illustration of the iTFC-S/M membrane’s production process [100].

Nanofiltration (NF) is favored for the removal of dye and large ions from aqueous media because of its exceptional efficacy, low power consumption, and environmental viability. Additionally, for the traditional thin-film composite (TFC) membrane, researchers have created novel materials for NF membranes. Many 2D nanomaterials, including carbon nitride (g-C_3_N_4_), GO, MXene, and metal–organic frameworks (MOFs), have drawn interest since single-layer graphene was found in 2004 [93,94,95]. Furthermore, 2D nanomaterials are more resilient to high temperatures and corrosive conditions than polymers. However, these layered materials’ organized interlayer channels and many nanopores allow for the quick and highly selective transit of water molecules [96]. 2D nanoparticles are more durable than polymers against the high heat and corrosive environments that NF may encounter. These layered materials’ organized interlayer channels and a large number of nanopores make them suitable for the efficient and selective transport of water molecules [97,98]. Because of their remarkable qualities, MXenes have attracted a lot of attention considering recent developments in water treatment [99]. The molecular weight threshold was tailored and the dye/salt separation effectiveness was increased by incorporating Ti_3_C_2_T_x_ nanolayers into the aramid nanofiber (ANF) membrane. A substantially incorporated MXene-ANF membrane (20 wt%) demonstrated notable refusal rates for organic compounds without sacrificing water permeability. Interestingly, at a salt concentration of 5 g/L, the most efficient nanocomposite membrane showed an insignificant rejection (0.2%) of NaCl while attaining rejection rates of around 99% for Alcian Blue, 94% for Rose Bengal, and 94% for Congo Red (CR). In the recently published report, Jiang et al., fabricate a unique method for intercalating sodium alginate (SA) into MXene nanosheets to control interfacial polymerization (IP). By increasing the interlayer distance between MXene nanosheets, SA increases the penetration area and lessens the vulnerability to swelling brought on by weak contacts between neighboring stacked nanosheets. The intermediate layer’s presence enhances permeability while efficiently controlling piperazine diffusion and encouraging the development of a full polyamide (PA) layer with ridge–valley structures (Figure 15). The constructed nanofiltration membrane maintains a rejection efficiency of 96.3% for divalent ions while achieving a permeability of 32.4 Lm^−2^h^−1^bar^−1^ [100]. In a different study conducted by Chen et al., the MXene-regulated interfacial polymerization (MRIP) technique was created to create high-performance polyamide (PA) NF membranes based on polyethyleneimine (PEI). The MXene-regulated membrane (PA@M) was more hydrophilic, had bigger pores, and was thinner. Consequently, the PA@M membrane’s permeability rose by almost 300 percent. Additionally, the membrane’s cation selectivity was exceptional, with >98% rejection for Mg^2+^ and <20% rejection for Li^+^ [101]. The recent trend of creating high-performance membranes to lower energy consumption and production costs for water treatment is in line with the integration of MXene nanosheets into PA nanofilm.

## 3. Conclusions and Future Perspective

The goal of this article is to give an overview of various techniques for scheming mix matrix membranes to enhance separation methods such as ultrafiltration, nanofiltration, membrane distillation, and reverse osmosis. The engineering of 2D nanostructured lamellar membranes has distinctly strengthened membrane permeability and selectivity, thus enhancing membrane performances. The development of MXene-based membranes with economical and environmentally sustainable approaches must be prioritized by researchers. Integration of Ti_3_C_2_T_x_ into RO membranes enhances water permeability, hydrophilicity, and salt rejection, with potential anti-fouling. Continued research on MXene-based membranes as a promising material is paramount for ensuring universal access to clean water and to address pressing global water challenges. Furthermore, in terms of maintaining the quality of these membranes, the scaling up of nanofiltration in industrial applications is still challenging and underdeveloped. In addition, it is of the utmost need to conduct research evaluations on the toxicity, biosafety, cytotoxicity, biocompatibility, storage conditions, life cycle, and potentially hazardous nature of these materials. Given membrane fabrication methods, blending/mixing the 2DMs with the polymer matrix to prepare MMMs is straightforward and effective in advancing the polymer membrane performance.

## Figures and Tables

**Table 1:** Water flux efficiency of the membranes prepared using different dopants for polyether sulfone membranes and polyvinylpyrrolidone.

Membrane Combination	Water Flux (L m^−2^h^−1^)	Reverse Salt Flux (g m^−2^h^−1^)	Reference
MXene + polyether sulfone + polyvinylpyrrolidone	12.05	8.43	[56]
Arginine + polyether sulfone	22.7	3.1	[57]
n-aminoethyl piperazine propane sulfonate + polyether sulfone	15	5.7	[58]
(1-(3-aminopropyl)-imidazole) propane-sulfonate + polyether sulfone	22.7	3.4	[59]
2-[(2-aminoethyl) amino]-ethane sulfonic acid monosodium salt + polyether sulfone	13.5	8.8	[60]
Cellulose acetate + TiO_2_ + polyvinyl pyrrolidone	58.21	16.28	[61]
